# Ascorbic Acid Supplementation Improves Skeletal Muscle Growth in Pacu (*Piaractus mesopotamicus*) Juveniles: In Vivo and In Vitro Studies

**DOI:** 10.3390/ijms22062995

**Published:** 2021-03-15

**Authors:** Bruna Tereza Thomazini Zanella, Isabele Cristina Magiore, Bruno Oliveira Silva Duran, Guilherme Gutierrez Pereira, Igor Simões Tiagua Vicente, Pedro Luiz Pucci Figueiredo Carvalho, Rondinelle Artur Simões Salomão, Edson Assunção Mareco, Robson Francisco Carvalho, Tassiana Gutierrez de Paula, Margarida Maria Barros, Maeli Dal-Pai-Silva

**Affiliations:** 1 Department of Structural and Functional Biology, Institute of Biosciences, São Paulo State University, UNESP, Botucatu 18618-689, São Paulo, Brazil; bruna.zanella@unesp.br (B.T.T.Z.); isabele.magiore@unesp.br (I.C.M.); guilherme.gutierrez@unesp.br (G.G.P.); robson.carvalho@unesp.br (R.F.C.); tassianagutierrez@gmail.com (T.G.d.P.); 2 Department of Histology, Embryology and Cell Biology, Institute of Biological Sciences, Federal University of Goiás (UFG), Goiânia 74690-900, Goiás, Brazil; brunoduran@ufg.br; 3 Department of Breeding and Animal Nutrition, School of Veterinary Medicine and Animal Science, São Paulo State University, UNESP, Botucatu 18618-681, São Paulo, Brazil; igor.tiagua@unesp.br (I.S.T.V.); pedro.pucci@unesp.br (P.L.P.F.C.); margarida.barros@unesp.br (M.M.B.); 4 Environment and Regional Development Graduate Program, University of Western São Paulo, Presidente Prudente 19050-680, São Paulo, Brazil; rondinellesalomao@gmail.com (R.A.S.S.); edsonmareco@gmail.com (E.A.M.)

**Keywords:** ascorbic acid, skeletal muscle growth, fasting, myogenesis, anabolism

## Abstract

In fish, fasting leads to loss of muscle mass. This condition triggers oxidative stress, and therefore, antioxidants can be an alternative to muscle recovery. We investigated the effects of antioxidant ascorbic acid (AA) on the morphology, antioxidant enzyme activity, and gene expression in the skeletal muscle of pacu (*Piaractus mesopotamicus*) following fasting, using in vitro and in vivo strategies. Isolated muscle cells of the pacu were subjected to 72 h of nutrient restriction, followed by 24 h of incubation with nutrients or nutrients and AA (200 µM). Fish were fasted for 15 days, followed by 6 h and 15 and 30 days of refeeding with 100, 200, and 400 mg/kg of AA supplementation. AA addition increased cell diameter and the expression of anabolic and cell proliferation genes in vitro. In vivo, 400 mg/kg of AA increased anabolic and proliferative genes expression at 6 h of refeeding, the fiber diameter and the expression of genes related to cell proliferation at 15 days, and the expression of catabolic and oxidative metabolism genes at 30 days. Catalase activity remained low in the higher supplementation group. In conclusion, AA directly affected the isolated muscle cells, and the higher AA supplementation positively influenced muscle growth after fasting.

## 1. Introduction

Skeletal muscle is a tissue that responds to different stimuli, and in fish, it represents approximately 60% of their body weight, being a vital source of amino acid [1,2]. Moreover, since skeletal muscle is the edible tissue of these animals, aquaculture production represents a great agronomic activity to satisfy human consumption [3]. Fish also represent a model to study skeletal muscle, since these animals can increase muscle mass both by adding new fibers, called the hyperplasia process, and by increasing the diameter of pre-existing fibers, referred to as the hypertrophy process. These characteristics highlight a contrast between fish and other animals as mammals, whereas amniotes do not present hyperplasia in juvenile and adult stages (reviewed in [4]).

Throughout their lives, fish are subjected, both in natural and artificial environments, to periods of food restriction that lead to muscle degradation [5,6]. In fasting situations, the balance of the processes that coordinate muscle maintenance is affected, increasing the degradation activity and the expression of catabolic genes, such as in the ubiquitin–proteasome system, while decreasing the synthesis signaling and the expression of anabolic genes, such as in the Igf1 pathway [7,8,9,10]. Moreover, the regulation of myogenic genes that promote muscle cell proliferation and differentiation and the systems related to energy production, such as oxidative metabolism, can also be affected by nutrient unavailability [11,12,13,14,15,16].

In aquaculture, given that muscle loss can affect the final profits, a search for strategies to encourage muscle recovery has been undertaken in different species. The central approach in this situation is the refeeding process, which can lead to compensatory growth. In this condition, refed fish can reach the size of non-fasted animals and can even surpass their weight and length [17]. Several refeeding periods have been tested as a strategy to promote muscle gain in species such as *Salmo salar* (Atlantic salmon) [14], *Oncorhynchus mykiss* (rainbow trout) [9,18,19], *Ctenopharyngodon idellus* (grass carp) [13], and *Piaractus mesopotamicus* (pacu) [11,20,21]. Additionally, the supplementation of growth factors, such as Igf1 and amino acids, has been applied in vivo in rainbow trout [22] and *Paralichthys adspersus* (fine flounder) [23], as well as in vitro in the muscle cells of Atlantic salmon [24], rainbow trout [25] and *Sparus aurata* (sea bream) [26]. Although these strategies are efficient in promoting muscle recovery, they might require several days to achieve total restoration [13,21,23].

Feed restriction can also increase oxidative stress and the production of reactive oxygen species (ROS) [27]. Animals have an internal antioxidant system composed of non-enzymatic and enzymatic agents. Enzymes such as superoxide dismutase (Sod), catalase (Cat), and glutathione peroxidase (Gpx) convert ROS into non-damage molecules (reviewed in [28]). In addition, external antioxidant sources can be added to assist ROS neutralization. In this context, antioxidants may prevent or stop an increase in ROS production [28]. Ascorbic acid (AA) is one of the most potent antioxidants and reduction agents [29]. It has crucial roles in ROS neutralization, a fundamental function in the defense against the toxicity and maintenance of cells’ redox state [29]. Moreover, AA can promote glutathione and vitamin E regeneration, which acts as an antioxidant protector in biological membranes [29]. Recently, the beneficial effects of AA have been described in skeletal muscle. In the absence of AA, aged rats experience an increase in ROS production, a reduction in muscle mass, and an impairment in physical performance [30]. Additionally, AA and vitamin E supplementation has been shown to decrease lipid peroxidation, DNA damage, and H_2_O_2_ production in aged rats submitted to repetitive loading exercises [31]. In isolated mammalian muscle cells, AA supplementation impacted cell differentiation [32] and myotube production [33].

The global value of aquaculture has ascended from 68.5 to 148.6 billion US dollars from 2000 until 2014 [34]. Only in South America, 12 million dollars were produced during this period, and 77% of the total was originated from Chile, Ecuador, and Brazil [34]. In Brazil, the production of “round” fishes, including the pacu, comprises about 30% of the national fisheries production [35]. The pacu has valuable characteristics for intensive cultivation as fast growth, great adaptation to different food sources, and continuous muscle growth [36,37,38]. Besides, from 2000 to 2014, it was the 14th most farmed species in South America [34]. Since fish do not produce AA endogenously [29,39], the significance of its supplementation in the normal development of the skeletal system, operculum, and gills has been proven [40]. AA also improved hematological, immunological, and growth parameters and promoted protection against genotoxicity and stressor agents in fish [41,42,43,44]. Despite the recent discovery of the actions of AA in proliferation, migration, and differentiation in pacu muscle cells treated with oxidant agent [45], this compound’s impact on the recovery of skeletal muscle in fish remains unexplored.

The impact of investigations using fish’s muscle cell culture has increased in the last years (reviewed in [46]). This approach, which restricts the systemic interactions between tissues, provided answers to important questions in the fish muscle physiology, as the action of external and internal growth factors during the myogenesis of isolated trout cells [18,47,48,49] and sea bream myocytes [50], besides the microRNAs participation in the myogenesis of isolated pacu muscle cells [51]. In vitro procedures were also recently described as a model to study slow muscle features in rainbow trout [52]. In this sense, the in vitro studies are a great complement to in vivo observations in fish muscle, being a remarkable strategy for the variables control [46].

Given this scenario, the present study evaluated the effects of AA supplementation on the morphology, antioxidant enzyme activity, and gene expression in the skeletal muscle of pacu following fasting, using both in vitro and in vivo strategies. We demonstrated that AA supplementation directly affects isolated muscle cells, thereby increasing the cell diameter and the expression of genes related to anabolism and cell proliferation. Moreover, high levels of AA increased the muscle fiber diameter and the expression of genes related to anabolism, cell proliferation, and oxidative metabolism, as well as decreased Cat activity, which positively influenced muscle growth resumption after food restriction.

## 2. Results

### 2.1. Fasting Followed by AA Supplementation Promotes Morphological Alterations in Skeletal Muscle In Vitro and In Vivo

**Figure 1:**
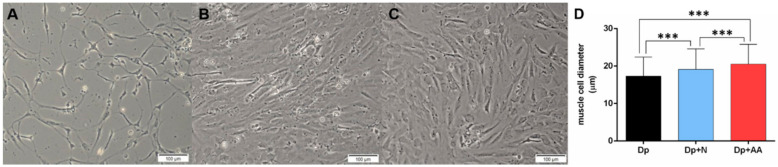
In vitro muscle cell diameter in conditions of nutrient deprivation (Dp) for 96 h, nutrient deprivation (72 h) + nutrient addition (24 h) (Dp + N), and nutrient deprivation (72 h) + AA supplementation (24 h) (Dp + AA). Representative images of pacu muscle cell culture in (**A**) Dp, (**B**) Dp + N, and (**C**) Dp + AA groups. Images obtained under an inverted microscope at a 10× magnification (Bars: 100 µm). (**D**) Muscle cell diameter in Dp, Dp + N, and Dp + AA groups. Data represented as mean ± SD. Statistical significance was determined by Kruskal-Wallis followed by Dunn’s post hoc test (*n* = 3 cell cultures/group). *** Denotes *p* < 0.001. Dp = deprivation; N = nutrient; AA = ascorbic acid.

Since in vitro studies allow the control of the environment by the modification of the culture media to evaluate the effect of a specific nutrient (reviewed in [53]), we isolated and cultivated muscle cells from pacu to understand if AA supplementation can affect muscle cell in nutrient restriction situation, regarding the influence of other tissues. We measured the diameter of cells using images obtained from an inverted microscope to evaluate the morphological impact of nutrient deprivation and AA supplementation in isolated muscle cells. Nutrient deprivation decreased the cell diameter in the Dp group (Figure 1A,D), which was reestablished after the addition of the growth medium in the Dp + N group (Figure 1B,D). AA supplementation (+ growth medium) resulted in cells with higher diameters in the Dp + AA group (Figure 1C,D), indicating that the deprivation impaired growth and the nutrients ameliorated this process, while the AA enhanced cellular muscle growth after nutrient deprivation.

**Figure 2:**
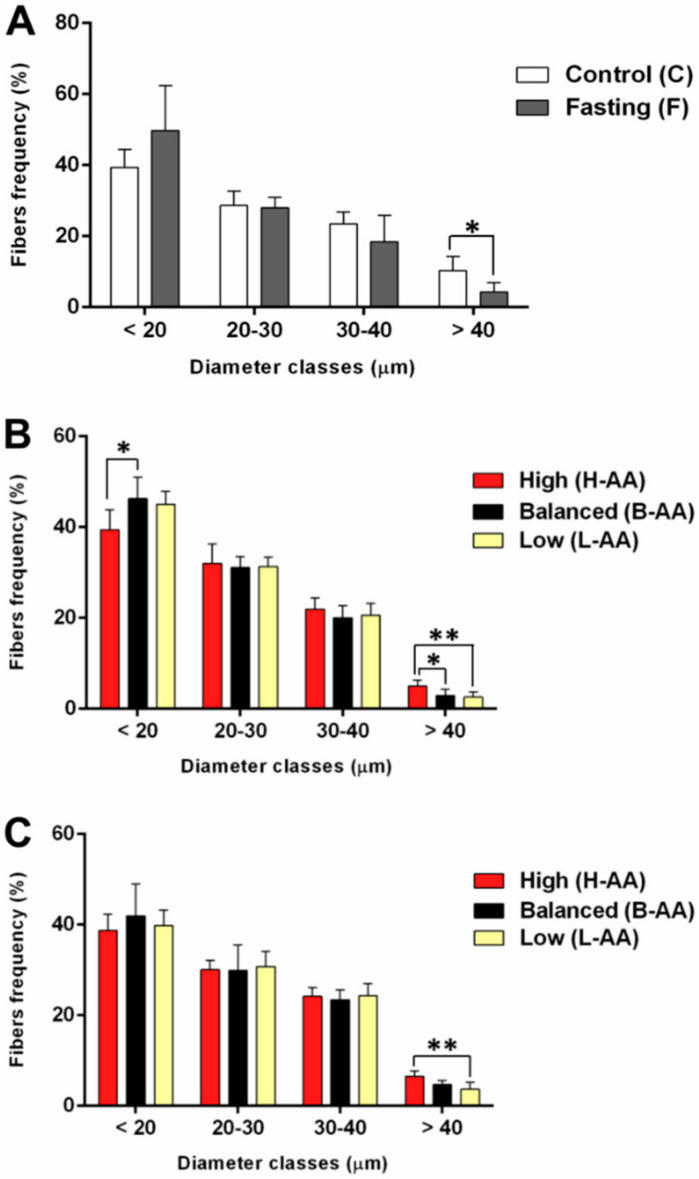
In vivo muscle fibers diameter in muscle tissue of pacus after (**A**) 15 days of fasting (F group) and continuous feeding (C group), and in pacus supplemented with AA in the concentration of 400 mg/kg (H-AA group), 200 mg/kg (B-AA group), and 100 mg/kg (L-AA group) for (**B**) 15 days and (**C**) 30 days of refeeding, after 15 days of fasting. Fibers were separated into classes according to their diameter. Data are represented as mean ± SD. Statistical significance was determined by Mann-Whitney between C and F groups and by one-way ANOVA followed by Tukey’s post hoc test between refeeding groups (*n* = 6 animals/group). * Denotes *p* < 0.05, ** denotes *p* < 0.01. AA = ascorbic acid.

We also evaluated the diameter of muscle fibers, in vivo, to confirm our fasting protocol’s ability to promote a reduction in muscle fiber area and to elucidate the impact of AA supplementation during refeeding. After fasting, there was a decrease in the frequency of fibers with a higher diameter (Figure 2A and Figure S1B). We also observed a reduction in body weight (Figure S2A) and length (Figure S2B). After 15 days of refeeding, we observed, in the higher supplementation group (H-AA), an increase in the frequency of fibers with a higher diameter in comparison to the balanced (B-AA) and low (L-AA) supplementation groups, as well as a decrease in the frequency of fibers with a smaller diameter in the H-AA related to B-AA group (Figure 2B). After 30 days, the H-AA group experienced an increased frequency of fibers with a higher diameter compared to the L-AA group (Figure 2C and Figure S1E), indicating that our fasting protocol led to muscle loss, whilst a higher AA supplementation increased the diameter of muscle fibers throughout the experimental period, reinforcing the in vitro results.

### 2.2. AA Supplementation Promotes Changes in Antioxidant Enzyme Activity In Vitro and In Vivo

**Figure 3:**
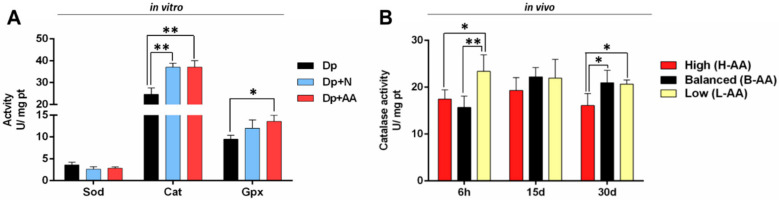
In vitro activity of superoxide dismutase (Sod), catalase (Cat), and glutathione peroxidase (Gpx) in (**A**) isolated muscle cells in conditions of nutrient deprivation (Dp) for 96 h, nutrient deprivation (72 h) + nutrient addition (24 h) (Dp + N), and nutrient deprivation (72 h) + AA supplementation (24 h) (Dp + AA). (**B**) In vivo Cat activity in muscle tissue of pacus submitted to 15 days of fasting and supplemented with 400 mg/kg (H-AA group), 200 mg/kg (B-AA group), and 100 mg/kg (L-AA group) of AA for 6 h, 15, and 30 days of refeeding after 15 days of fasting. Data represented as mean ± SD. Statistical significance was determined by one-way ANOVA followed by Tukey’s post hoc (*n* = 4 cell culture/group, in vitro; *n* = 6 animals/group, in vivo). * Denotes *p* < 0.05, ** denotes *p* < 0.01. Dp = deprivation; N = nutrient; AA = ascorbic acid.

Considering the stressful effects of nutrient deprivation and fasting [27] and the antioxidant actions of AA [29], we evaluated antioxidant enzyme activity. In vitro nutrient deprivation decreased Cat activity in the Dp group compared to the Dp + N and Dp + AA groups. It also decreased Gpx activity in the Dp group compared to the Dp + AA group (Figure 3A). In our in vivo experiment, fasting did not change the antioxidant enzyme activity (Figure S3A). The B-AA and H-AA groups experienced a decrease in Cat activity after 6 h of refeeding in comparison to the L-AA group, which was sustained at 30 days only in the H-AA group (Figure 3B). AA supplementation did not affect Sod and Gpx activities (Figure S3B,C). It is possible that AA could differently affect the activity of antioxidant enzymes, considering the animal conditions and the experimental model, possibly maintaining a favorable antioxidant microenvironment.

### 2.3. AA Supplementation Affects Genes Expression In Vitro and In Vivo

#### 2.3.1. Expression of Anabolic Genes Increases with AA Supplementation

**Figure 4:**
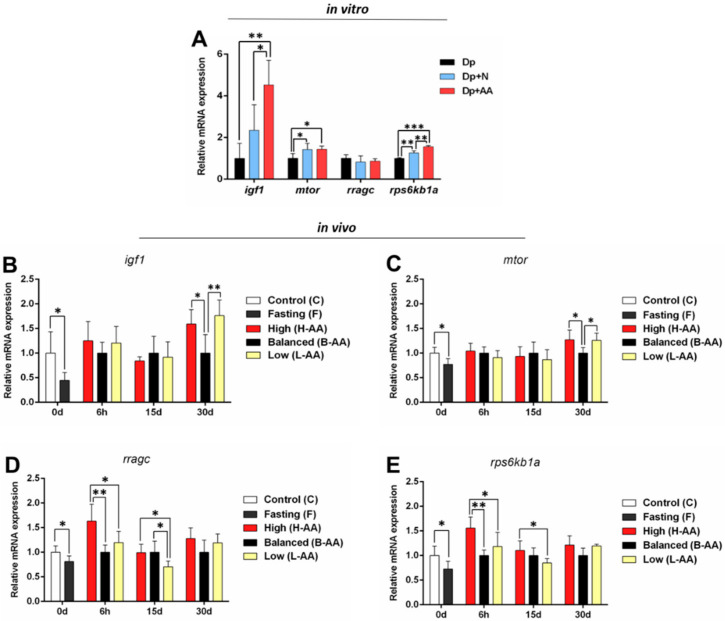
Anabolic genes expression. (**A**) In vitro expression of *igf1, mtor, rragc* and *rps6kb1a* in isolated muscle cells in conditions of nutrient deprivation (Dp) for 96 h, nutrient deprivation (72 h) + nutrient addition (24 h) (Dp + N), and nutrient deprivation (72 h) + AA supplementation (24 h) (Dp + AA). Fold change from the Dp group. Relative mRNA expression normalized to *rpl13* expression. In vivo genes expression during fasting (0 d) and refeeding with AA supplementation. (**B**) *igf1*, (**C**) *mtor*, (**D**), *rragc,* and (**E**) *rps6k1a* expression in muscle tissue of pacus continuous fed (C group) and submitted to 15 days of fasting (F group) and in pacus supplemented with 400 mg/kg (H-AA group), 200 mg/kg (B-AA group), and 100 mg/kg (L-AA group) of AA for 6 h and 15 and 30 days of refeeding after 15 days of fasting. Fold change from the B-AA group. Relative mRNA expression normalized to *rpl13* expression. Data represented as mean ± SD. Statistical significance was determined by unpaired T-test between C and F groups and by one-way ANOVA followed by Tukey’ post hoc test between refeeding groups and in in vitro experiments (*n* = 5 cell culture/group, in vitro; *n* = 6 animals/group, in vivo). * Denotes *p* < 0.05, ** denotes *p* < 0.01, *** denotes *p* < 0.001. Dp = deprivation; N = nutrient; AA = ascorbic acid.

We first evaluated the expression of the genes from Igf1 signaling pathway, which is known to modulate skeletal muscle growth (reviewed in [54]). Nutrient deprivation decreased the expression of several anabolic genes, such as *igf1, mtor,* and *rps6kb1a*, in vitro (Figure 4A), and fasting reduced the expression of *igf1, mtor, rragc,* and *rps6kb1a* in vivo (Figure 4B–E). In vitro AA supplementation increased the expression of *igf1* and *rps6kb1a* in the Dp + AA group compared to the Dp and Dp + N groups. *rps6kb1a* expression also increased in the Dp+N group compared to the Dp group; however, it was lower compared to the Dp + AA group. Moreover, both the Dp + N and Dp + AA groups showed increased expression of *mtor* in comparison to the Dp group (Figure 4A), indicating that nutrients as amino acids can directly stimulate *mtor* expression [26] but that the action of AA may enhance the anabolic activity.

After 6 h of refeeding, we found an increase in *rragc* and *rps6kb1a* expression in the H-AA group compared to the other refeeding groups (Figure 4D,E). After 15 days, the L-AA group had a decrease in the expression of *rragc* in comparison to the B-AA and H-AA groups and in *rps6kb1a* compared to the H-AA group (Figure 4D,E). *igf1* and *mtor* expression decreased in the B-AA group in comparison to the L-AA and H-AA groups after 30 days of refeeding (Figure 4B,C). Therefore, higher AA supplementation may stimulate the expression of anabolic genes at the beginning of the refeeding protocol, while low supplementation may impact this process even after 15 days of refeeding.

#### 2.3.2. Catabolic Gene Expression Is Modulated Throughout the Experimental Protocols

**Figure 5:**
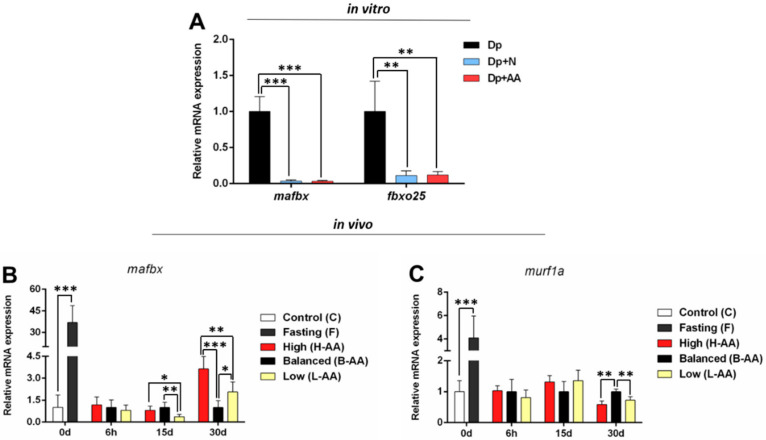
Catabolic genes expression. (**A**) In vitro *mafbx* and *fbxo25* expression in isolated muscle cells in conditions of nutrient deprivation (Dp) for 96 h, nutrient deprivation (72 h) + nutrient addition (24 h) (Dp + N) and nutrient deprivation (72 h) + AA supplementation (24 h) (Dp + AA). Fold change from the Dp group. Relative mRNA expression normalized to *rpl13* expression. In vivo genes expression during fasting (0 d) and refeeding with AA supplementation. (**B**) *mafbx* and (**C**) *murf1a* expression in muscle tissue of pacus continuous fed (C group) and submitted to 15 days of fasting (F group) and in pacus supplemented with 400 mg/kg (H-AA group), 200 mg/kg (B-AA group), and 100 mg/kg (L-AA group) of AA for 6 h and 15 and 30 days of refeeding after 15 days of fasting. Fold change from B-AA group. Relative mRNA expression normalized to *rpl13* expression. Data represented as mean ± SD. Statistical significance was determined by unpaired T-test between C and F groups and by one-way ANOVA followed by Tukey’ post hoc test between refeeding groups and in in vitro experiments (*n* = 5 cell culture/group, in vitro; *n* = 6 animals/group, in vivo). * Denotes *p* < 0.05, ** denotes *p* < 0.01, *** denotes *p* < 0.001. Dp = deprivation; N = nutrient; AA = ascorbic acid.

The ubiquitin–proteasomal system has a significant catabolic function in muscle, contributing to muscle degradation and turnover [55]. Our in vitro and in vivo protocols of nutrient restriction increased the expression of catabolic genes, such as *mafbx* and *fbxo25* in vitro (Figure 5A), as well as *mafbx* and *murf1a* in vivo (Figure 5B,C), indicating a muscle loss response in this condition. In the nutrient and AA media, *mafbx* and *fbxo25* expression decreased (Figure 5A), indicating a reduction in the catabolic process. In vivo, after 15 days of refeeding, *mafbx* expression declined in the L-AA group compared to the other refeeding groups (Figure 5B). At 30 days, the H-AA group had higher *mafbx* expression related to the other refeeding groups, and the L-AA group experienced an increase in the expression of this gene compared to the B-AA group (Figure 5B). Moreover, *murf1a* expression decreased in the L-AA and H-AA groups at this time (Figure 5C). Our findings indicate a temporal modulation of catabolic genes throughout the experimental period, which was affected, in vivo, by the amount of AA in the meal.

#### 2.3.3. Myogenic Genes May Contribute to Muscle Recovery in Response to AA Supplementation

**Figure 6:**
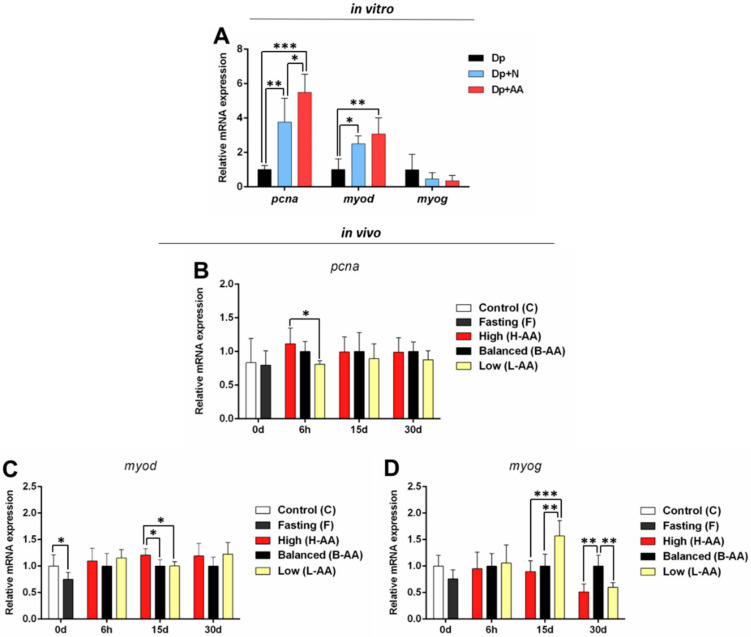
Expression of genes related to cell proliferation and differentiation. (**A**) In vitro *pcna, myod,* and *myog* expression in isolated muscle cells in conditions of nutrient deprivation (Dp) for 96 h, nutrient deprivation (72 h) + nutrient addition (24 h) (Dp + N), and nutrient deprivation (72 h) + AA supplementation (24 h) (Dp + AA). Fold change from the Dp group. Relative mRNA expression normalized to *rpl13* expression. In vivo genes expression during fasting (0 d) and refeeding with AA supplementation. (**B**) *pcna*, (**C**) *myod,* and (**D**) *myog* expression in muscle tissue of pacus continuous fed (C group) and submitted to 15 days of fasting (F group) and in pacus supplemented with 400 mg/kg (H-AA group), 200 mg/kg (B-AA group), and 100 mg/kg (L-AA group) of AA for 6 h and 15 and 30 days of refeeding after 15 days of fasting. Fold change from B-AA group. Relative mRNA expression normalized to *rpl13* expression. Data represented as mean ± SD. Statistical significance was determined by unpaired T-test between C and F groups and by one-way ANOVA followed by Tukey’ post hoc test between refeeding groups and in in vitro experiments (*n* = 5 cell culture/group, in vitro; *n* = 6 animals/group, in vivo). * Denotes *p* < 0.05, ** denotes *p* < 0.01, *** denotes *p* < 0.001. Dp = deprivation; N = nutrient; AA = ascorbic acid.

Myogenic genes are modulators of muscle development in the initial developmental stages and contribute to muscle growth in the post-natal phases controlling cell proliferation and differentiation [56,57]. Nutrient deprivation decreased *pcna* expression in vitro (Figure 6A) and *myod* expression both in vitro (Figure 6A) and in vivo (Figure 6C), indicating impaired muscle cell proliferation. In vitro *pcna* expression increased in the Dp + N related to Dp group, while the Dp + AA group presented the higher expression of this gene (Figure 6A). In vivo, at 6 h of refeeding, the *pcna* expression increased in the H-AA related to L-AA group (Figure 6B), and *myod* expression was higher in the H-AA in comparison to other refeeding groups at 15 days of refeeding (Figure 6C). Additionally, at this point, the B-AA and H-AA groups experienced decreased *myog* expression (Figure 6D). At 30 days, the L-AA and H-AA groups also experienced decreased *myog* expression (Figure 6D). These findings indicate that high AA levels stimulate cell proliferation and discourage cell differentiation.

#### 2.3.4. Changes in the Expression of Mitochondrial Genes Contribute to Muscle Maintenance during Fasting and Recovery during Refeeding

**Figure 7:**
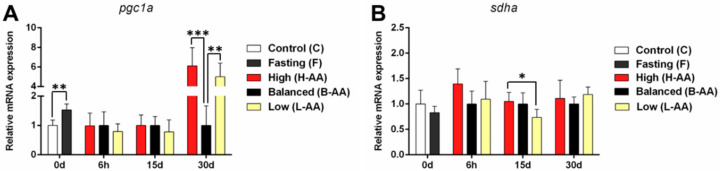
In vivo oxidative metabolism genes expression during fasting (0d) and refeeding with AA supplementation. (**A**) *pgc1a* and (**B**) *sdha* expression in muscle tissue of pacus continuous fed (C group) and submitted to 15 days of fasting (F group) and in pacus supplemented with 400 mg/kg (H-AA group), 200 mg/kg (B-AA group), and 100 mg/kg (L-AA group) of AA for 6 h and 15 and 30 days of refeeding after 15 days of fasting. Fold change from the B-AA group. Relative mRNA expression normalized to *rpl13* expression. Data represented as mean ± SD. Statistical significance was determined by one-way ANOVA followed by Tukey’s post hoc test (*n* = 6 animals/group). * Denotes *p* < 0.05, ** denotes *p* < 0.01, *** denotes *p* < 0.001. AA = ascorbic acid.

Mitochondrial metabolism contributes to energy generation in different situations, and it is the primary source of ROS production [58]. Although our in vitro protocol did not promote alterations in the expression of *pgc1a*, which is translated into a co-factor associated with mitochondrial biogenesis [59] (Figure S4), the fasting period increased the expression of this gene in vivo (Figure 7A), indicating enhancement in alternative energy production in the absence of food. At 15 days of refeeding, *sdha* expression decreased in the L-AA group compared to the H-AA group (Figure 7B). At 30 days, *pcg1a* expression increased in the L-AA and H-AA groups (Figure 7A). The increased *sdha* and *pgc1a* expression the H-AA group indicates the maintenance of muscle oxidative metabolism throughout the experimental period with higher AA supplementation.

## 3. Discussion

Skeletal muscle is mainly composed of proteins. In addition to structural and contractile functions, these proteins can be degraded and used as a source of amino acid during food restriction conditions [7]. Our study demonstrated decreased muscle cell and muscle fiber diameters and increased catabolic gene expression during nutrient deprivation and fasting situations in pacu. After high AA supplementation, we found an increase in pacu muscle cell and muscle fiber diameters, in vitro and in vivo increased anabolic and myogenic gene expression, and increased expression of mitochondrial genes combined with decreased Cat activity in vivo.

Fasting periods can happen due to factors such as migration, reproduction, and food shortages, and it is commonly found at some stage throughout the lifetime of different fish species. Metabolic reserve mobilization allows the survival of animals in response to this challenge [5]. In this work, the muscle cells cultured in deprivation medium (Dp group) had a decrease in diameter, an increase in catabolic genes expression, a reduction in anabolic and myogenic genes expression, and in antioxidant enzyme activity. Furthermore, our fasting protocol was confirmed in vivo by a decline in the diameter of the muscle fibers, as well as in the body weight and length of the fish. Moreover, we observed increased catabolic and decreased anabolic gene expression in these animals. This profile of alterations is well characterized and allows animal survival by inhibiting processes with energy costs, such as anabolism, and by stimulating processes that provide energy, such as catabolism and oxidative metabolism (reviewed in [7]). Similar results have been found in other fish species subjected to different fasting protocols, such as *Oreochromis niloticus* (Nile tilapia) [15], grass carp [13], as well as in both in vivo and in vitro experiments in Atlantic salmon [14,24], gilthead sea bream [26], and rainbow trout [60]. These alterations have also been observed in pacu in different development phases [11,20,21], reinforcing the nutrient deprivation and fasting protocols applied in the present work as a strategy for muscle remodeling. Besides, a coordinate transcriptional response during fasting conditions is also observed in hibernating mammals. During torpor and hibernation, although the expression of genes related to protein degradation increases, as observed in bears and bats ([61], reviewed in [62]), it seems that both transcriptional and translational activity is generally depressed in several pathways, which may result in the maintenance of muscle mass (reviewed in [62]). This condition is in line with the findings observed in the present work that may indicate an overall depression of anabolic transcriptional activity in fasting situation in pacus.

ROS production typically occurs in aerobic organisms mainly due to mitochondrial metabolism, and in high concentrations, these molecules can cause cellular damage [58]. Conditions as food restriction can trigger an increase in ROS production in cells [27]. Even though the antioxidant enzyme activity did not change in the fasted animals, our nutrient deprivation and fasting protocols may have promoted an increase in oxidative stress, thereby affecting muscle cells and tissue maintenance, since AA supplementation resulted in interesting changes.

Vitamin C, or AA, is considered one of the most effective antioxidant agents that can inhibit a series of harmful molecules [29]. In the present study, at the beginning of the refeeding period (6 h), we did not find differences associated with different levels of AA supplementation in the expression of the genes related to muscle catabolism (*mafbx* and *murf1a*), myogenesis (*myod* and *myog*), and oxidative metabolism (*pgc1α* and *sdha*). These results may be related mainly to the refeeding process than to the AA supplementation, since the expression of those genes were similar between the refeeding groups, and the feeding can modulate the expression of genes of different signaling pathways even in the initial hours of refeeding as seen in Atlantic salmon [14] and Chinese perch (*Siniperca chuatsi*) [63].

While the expression of some genes was not affected by the AA level, we found interesting changes in the expression of anabolic genes at 6 h of refeeding. Although the expression of the *igf1* and *mtor* was similar between the groups, which is probably related to the moment of evaluation considering the dynamic gene expression in signaling pathways and that the initial transcriptional response can happen in minutes (reviewed in [64]), we found increased expression of the critical components of anabolic signaling, i.e., *rragc* and *rps6kb1a*, only in the group that was supplemented with the higher amount of AA (H-AA group).

The *rragc* gene codifies a protein that compounds the heterodimer RagA/RagB–RagC/RagD, which in the presence of amino acids can stimulate protein synthesis through the allocation of the *mtorc1* complex near the lysosomal membrane (reviewed in [65]). The silencing of RagB and RagC in *Drosophila* cells leads to a decrease in the phosphorylation of Mtorc1 targets [66]. The *rps6kb1a* gene is responsible for coding the translation of a ribosomal kinase that acts in protein synthesis, and it is one of the main Mtorc1 targets (reviewed in [67]). An increase in the expression of this gene after feeding resulted in muscle growth in rainbow trout [18], and the inhibition of Mtor and, consequently, its downstream targets resulted in impaired muscle growth in fine flounder [8]. Considering the action of these molecules in muscle maintenance, the increase in *rragc* and *rps6kb1a* expression in the H-AA group may indicate a faster response to food consumption and a stimulus for protein synthesis, a process possibly reinforced by AA supplementation. Additionally, the decrease in Cat activity in the B-AA and H-AA groups at this timepoint supports our hypothesis of the antioxidant action of AA in skeletal muscle. Cat, an antioxidant enzyme, acts in ROS neutralization, turning it into non-reactive molecules (reviewed in [28]). It is possible that the low Cat activity in these groups could be a result of ROS neutralization by AA, since the substrate amount directly affects enzymatic activity [68,69].

The faster response in the H-AA group at 6 h of refeeding may have influenced the muscle phenotype at 15 days. At this time, we observed that the H-AA group had a decrease in the frequency of muscle fibers with a small diameter and an increase in the frequency of muscle fibers with a higher diameter. Moreover, we observed a general decrease in the expression of anabolic, catabolic, cell proliferation, and mitochondrial metabolism genes in the L-AA group. In addition to the elevated antioxidant enzyme activity in this group throughout the experimental period, this fact may indicate a prolonged effect of fasting, affecting the metabolic process and the recovery of the muscle mass with the lower AA level. In accordance, *Umbrina cirrosa* subjected to fasting has been shown to experience a decrease in general metabolic activity in skeletal muscle and a decrease in muscle mass [70]. Besides, mammals also experience an overall transcriptional depression during torpor and hibernation (reviewed in [62]), in line with the scenario observed in the present work with low AA supplementation.

Interestingly, we observed increased expression of *pcna* and *myod*, genes highly expressed in proliferative fish muscle cells ([71], reviewed in [3]), in the H-AA group at 6 h and 15 days of refeeding, respectively. In fish, the proliferation and differentiation of myoblasts contribute to muscle growth by incorporating their nuclei into nascent and expanding fibers [72]. Thus, the increased *pcna* and *myod* expression could indicate an improvement in muscle cell proliferation in response to refeeding and, possibly, due to the favorable redox status held by AA supplementation. Since these processes synergically contribute to muscle growth [73], the increased expression of the proliferative (*pcna* and *myod*) and anabolic genes (*rragc* and *rps6kb1a)* may explain our morphological results observed in the H-AA group at 15 days of refeeding. In fact, in our in vitro results, although in the absence of AA (Dp + N group), the cells showed improvements in the anabolic and hypertrophic processes, the supplemented cells showed higher diameters; higher expression of *igf1*, *rps6kb1a,* and *pcna*; and increased Gpx activity, indicating an even better and faster re-establishment of growth both by protein synthesis and cell proliferation. Similar results were found in pacu muscle cells cultivated with AA since isolation, showing that under both stressful or normal growth conditions, the antioxidant increased anabolic and decreased catabolic gene expression [45]. Moreover, AA and Igf1 supplementation restored the myogenic process in mice C2C12 and human myoblasts cultured at low temperatures [32]. Therefore, it is possible that in our study, the myogenic and anabolic response was late in the L-AA and B-AA groups, as 30 days of refeeding were necessary to incur an increase in the frequency of muscle fibers with a higher diameter compared to the H-AA group.

The decreased *myog* expression, a gene related to muscle cell differentiation [72], in the B-AA and H-AA groups, could indicate an extension in the proliferation process promoted by AA supplementation. Similar findings have been observed in muscle cells of rainbow trout treated with anthocyanidins (antioxidants compounds), in which the molecular results indicated a delay in the cell cycle progression, although these cells were committed to terminal differentiation [74]. Furthermore, in our study, low *myog* expression was sustained at 30 days in the H-AA group, supporting our hypothesis of AA increasing muscle cell proliferation and delaying the cell differentiation rate.

At 30 days of refeeding, we found increased *pgc1a* expression, a mitochondrial biogenesis inductor [59], in the H-AA group. The increased expression of this gene combined with the low Cat activity may indicate that higher AA supplementation promoted, through ROS neutralization, a favorable microenvironment for energy production, providing better growing conditions. Mitochondrial energy production as an alternative energy source has been seen in fasting situations, as shown in the present work and similar fasting protocols [11,12]. We also found increased *mafbx* expression at the end of the experimental period in this group. Proteolysis is a process that occurs even under normal conditions, being required for muscle function and protein turnover (reviewed in [7,55]). Mice subjected to chronic loading increase their muscle growth rate, which is associated with increased proteasome system activity [75]. After *mafbx* silencing, the females animals decrease their muscle mass, indicating its action in muscle growth and remodeling [75]. Thus, the *mafbx* expression in the H-AA group at 30 days of refeeding could indicate the contribution of this gene through protein turnover to the hypertrophy process initiated at the beginning of refeeding and sustained at this time.

Surprisingly, considering the final experimental period, the L-AA group showed some molecular similarities to the H-AA group. In nutrient restriction situations, fish can increase food ingestion as a strategy to supply energetic needs. This behavior can recover the weight, length, and muscle growth parameters [17,76,77]. Increased food consumption was observed in pacus fed with vitamin C- and E-deficient diets, although the higher ingestion did not result in better growth rates [78]. Rainbow trout subjected to protein restriction show increased feed intake that results in growth, albeit smaller than the growth rate in non-restricted animals [79]. Similarly, grass carp subjected to fasting increase their feed intake during refeeding, which resulted in higher expression of the myogenic genes and decreased expression of the negative regulators of muscle growth [13]. Therefore, the similarities between the L-AA and H-AA groups in the expression of *igf1, mtor, murf1a, myog*, and *pgc1α* may be related to higher food consumption in the L-AA group during refeeding. Although the food supply was the same between the groups, the consumption was not measured, which could be a limitation of the present work. Despite this fact, our molecular, enzymatic, and morphological results indicate that a higher amount of food could be needed in the absence of AA to stimulate anabolic processes.

In summary, we demonstrated that higher AA supplementation improves anabolic, myogenic, and oxidative metabolism gene expression, as well as antioxidant enzymatic activity, thereby enhancing pacu muscle growth in the initial phases of refeeding in vivo. Moreover, we confirmed that AA supplementation also positively affects the growth of isolated muscle cells. Our findings may represent a strategy in aquaculture systems to enable faster muscle recovery after stressful conditions, thus reducing the costs of this situation and improving the final profits.

## 4. Materials and Methods

### 4.1. Experimental Conditions

The experiments were conducted according to the ARRIVE guidelines [80] and the National Council for the Control of Animal Experimentation (CONCEA) Brazilian legislation. The protocol was approved by the Ethics Committee on Use of Animals (CEUA, protocol number 1050) of the Institute of Biosciences, São Paulo State University (UNESP), Botucatu, São Paulo, Brazil. Juvenile pacus (3 months-old; 28.5 g ± 8.8) were obtained from the University of Western São Paulo (UNOESTE), Presidente Prudente, São Paulo, Brazil and cultivated at the Agribusiness Technology Agency (APTA), Presidente Prudente, São Paulo, Brazil. Fish were farmed in distinct recirculating systems in 0.25 m^3^ tanks with 35 fish in each tank (density of 140 fish/m^3^) at 28 °C under a 12 h light/12 h dark photoperiod. The experimental groups were kept in independent systems to avoid AA cross contaminations in the water. Each group was kept in three tanks, resulting in three replicates per groups.

**Figure 8:**
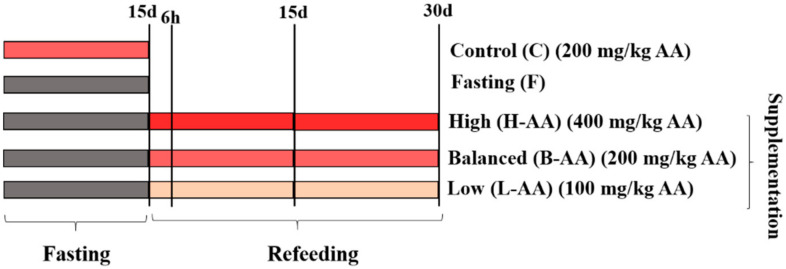
Experimental design used in our in vivo experiment. Initially, fish were divided in Control (C—continuous feeding) and Fasting (F—submitted to 15 days of fasting) groups. Subsequently, fish were submitted to refeeding with AA supplementation in concentration of 400 mg/kg (H-AA group), 200 mg/kg (B-AA group), and 100 mg/kg (L-AA group). Fast muscle samples were collected after 15 days of fasting and 6 h and 15 and 30 days of refeeding from 6 animals per experimental group.

Fish were fed ad libitum, three times daily with diets described in Table S1. To ensure the AA activity, the meals were prepared with polyphosphated vitamin C, protected against heating (Rovimix^®^, Stay-C^®^35, DSM Nutritional Products, Basel, Switzerland, 35% AA activity). The AA amounts in each diet were established following previous work using the same fish species [81,82,83]. The animals were subjected to an adaptation period of 15 days, which the meal had no AA supplementation. After, fish were divided into two groups: Control (C)—continuous feeding with AA in 200 mg/kg body weight—and Fasting (F)—food restriction for 15 days. After the fasting period, the fish were separated into three groups that received different AA amounts for 30 days: High-supplementation—400 mg/kg of AA supplementation; Balanced-supplementation (B-AA)—200 mg/kg of AA supplementation; and Low-supplementation (L-AA)—100 mg/kg of AA supplementation (Figure 8). Benzocaine at a concentration >250 mg/L was used to euthanize the animals. Body weight and length were measured, and white (fast) muscle samples were collected from the epaxial region, near the dorsal fin, after 15 days of fasting and after 6 h, 15 days, and 30 days of refeeding.

### 4.2. Primary Muscle Cell Culture

**Figure 9:**
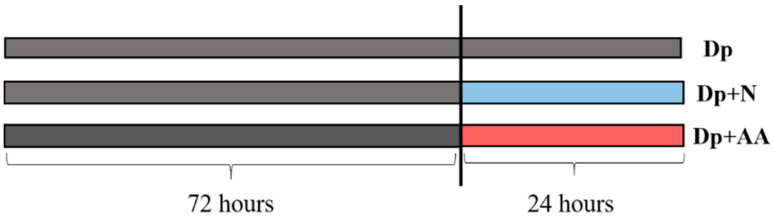
Isolated muscle cells from pacu were incubated with nutrient deprivation media for 96 h (Dp group); nutrient deprivation for 72 h followed by nutrient addition for 24 h (Dp + N group); and nutrient deprivation for 72 h followed by nutrient and AA addition for 24 h (Dp + AA groups). At the end of the experimental protocol, 96 h, the total RNA was extracted from 5 independent cell culture/group; the total proteins were extracted from 4 independent cell cultures/groups, and muscle cell diameters were assessed from 3 independent cell cultures/groups.

To complement our in vivo experiments, we isolated muscle cells from pacu to understand if AA supplementation would be able to act in muscle cells, regarding the influence of other tissues. The muscle cell isolation was conducted as previously described in Bower and Johnston [84] and other works with pacu [45,51]. Briefly, fast muscle samples were collected from 10–15 fish and, after mechanical and enzymatic digestion, cells were washed, counted, and seeded at a density of 1 × 10^6^ cells/mL in twelve well plates, previously treated with poly-L-lysine and laminin (Sigma-Aldrich, Saint Louis, MO, USA). Cells were cultured at 28 °C in complete growth medium (DMEM, 9 mM NaHCO_3_, 20 mM HEPES, 10% fetal bovine serum (FBS), and 1% antibiotics; Sigma-Aldrich, Saint Louis, MO, USA) for 10 days until they reached 80–90% confluency. Next, cells were divided into the groups: Dp—cultured for 96 h in deprivation medium (Earle’s balanced salt solution, supplemented with 9 mM NaHCO_3_, 20 mM HEPES, 2 g/L glucose, 1× vitamins, 1× antibiotics; Sigma-Aldrich, Saint Louis, MO, USA, adapted from [24]); Dp + N—cultured for 72 h in deprivation medium and 24 h in the complete medium; and Dp + AA—cultured for 72 h in deprivation medium and 24 h in complete medium supplemented with L-ascorbic acid 2-phosphate (200 µM) (Sigma-Aldrich, Saint Louis, MO, USA) (Figure 9), adapted from previous works with muscle cells [32,33,45].

### 4.3. Morphological Analyzes

For in vivo determination of muscle fibers diameter, histological muscle cryosections (10 µm) were obtained from fast muscle tissue of pacu (*n* = 6 animals/per group) in a cryostat and stained with hematoxylin and eosin. Images were obtained from six animals per group. The area of eight hundred fibers per animal was measured in ImageJ^®^ [85], and the fiber diameter was calculated following the method previously described [86]. Muscle fibers were grouped into classes according to their diameter [87,88]. For the determination of in vitro muscle cell diameter, images were obtained at the end of the experimental period from each group (n: 3 cell culture/per group) in an inverted microscope (Zeiss, Jena, Germany). The diameter was calculated using the mean of three measurements per cell, and one hundred cells per group were measured in ImageJ^®^ [85], adapted from [89,90]. Three independent isolation cell processes were performed to measure muscle cell diameter.

### 4.4. Gene Expression Analyzes

The total RNA was extracted from fast muscle tissue in vivo (*n* = 6 animals/per group) and muscle cells in vitro (*n* = 5 independent cell cultures/per group) using Trizol and following the manufacturer recommendations (Invitrogen (ThermoFisher Scientific, Carlsbad, CA, USA)). The RNA was quantified in the spectrophotometer NanoVue™ Plus (GE Healthcare, NJ, USA), and the integrity was ensured through electrophorese in 1% agarose gel. To avoid DNA contamination, the samples were treated with the RQ1 RNase-Free DNase kit (Promega, Madison, WI, USA) following the manufacturer recommendations. The total of 1 microgram/microliter of RNA was used to the synthesis of the cDNA, which was performed using the GoScript™ Reverse Transcription System (Promega, Madison, WI, USA) following the protocol recommendations. Gene expression levels were detected by RT-qPCR through QuantStudio™ 12 K Flex Real-Time PCR System (Applied Biosystems (ThermoFisher Scientific, Carlsbad, CA, USA) using the GoTaq^®^ qPCR Master Mix kit (Promega, Madison, WI, USA)) and primers (Table S2) designed from pacu’s transcriptome available in the European Nucleotide Archive, accession number PRJEB6656 [20]. Reactions were performed in duplicates, and the expression of genes related to myogenesis (*pcna*, *myod*, *myog*), anabolism (*igf1*, *mtor*, *rragc*, *rps6kb1a*), catabolism (*mafbx*, *murf1a*, *fbxo25*), and oxidative metabolism (*sdha, pgc1α*) was evaluated. The comparative Ct methods, 2^∆∆*C*t^ [91], were applied to calculate the gene expression levels, and the *rpl13* gene was selected as reference due to its stability and similarity between the groups.

### 4.5. Antioxidant Enzymes Activity

The total protein was extracted in vivo from fast muscle samples (*n* = 6 animals/per group) and in vitro muscle cells (*n* = 4 independent cell cultures/per group) in an extraction medium (Tris 50 mM, NaCl 0.2 M, Triton X-100 0.1%, CaCl_2_ 10 mM, Sigma-Aldrich, Saint Louis, MO, USA) with protease inhibitor (Sigma-Aldrich, Saint Louis, MO, USA). The samples were centrifuged for 20 min at 4 °C and 1968× *g*. The supernatant was collected and quantified following the Bradford method [92], using bovine serum albumin as reference.

To enzymatic activity analysis, all the reagents were purchased from Sigma Aldrich, Saint Louis, MO, USA, unless state otherwise. The Sod (EC 1.15.1.1) activity was measured following the Beauchamp and Fridovich method [93]. Briefly, phosphate buffer (pH 7.8), nitroblue tetrazolium (NBT, 33 mmol/L), EDTA (0.66 mmol/L), methionine (10 mmol/L), and riboflavin (0.0033 mmol/L) were added in tubes containing the protein samples at a concentration of 6 microgram/microliter. The antioxidant activity was measured in a spectrophotometer at 560 nm.

The Cat (EC 1.11.1.6) activity was determined through the reduction of the dichromate diluted in acetic acid to chromic acetate in H_2_O_2_ presence, as previously described in [94]. Samples at a concentration of 6 microgram/microliter were added to a mixture of H_2_O_2_ and potassium dichromate diluted in acetic acid. The activity was measured in a spectrophotometer at 620 nm.

The Gpx (EC 1.11.1.9) activity was quantified, as described in [95]. Phosphate buffer (pH 7.4), reduced glutathione (2 mM), and H_2_O_2_ were added to samples at 3 microgram/microliter concentration. The mixture was heated at 37 °C for 15 min, and 5% trichloroacetic acid was added. Next, the samples were centrifuged for 5 min at 327× *g*, and the supernatant was collected. For each 100 µL of the collected supernatant, 700 µL of DTNB (0.4 mg/mL) were added. The activity was measured in a spectrophotometer at 320 nm. The results of the activity of the antioxidant enzymes were expressed as U.mg pt^−1^.

### 4.6. Statistical Analyzes

For in vitro analyzes, the statistical significance related to gene expression and antioxidant enzyme activity was assessed using one-way ANOVA followed by Tukey’s post hoc test. For the differences related to muscle cells’ diameter, the Kruskal–Wallis followed by Dunn’s post hoc test was performed. For in vivo analyzes, an unpaired T-test was performed to assess the statistical significance between C and F groups in gene expression, antioxidant enzyme activity, and length. The statistical differences between these groups in weight and muscle fibers diameter were assessed using the Mann-Whitney test. After refeeding, the statistical significance related to body length and weight was tested, performing the Kruskal-Wallis followed by Dunn’s post hoc test. One-way ANOVA was followed by Tukey’s post hoc test to assess the statistical differences in gene expression, antioxidant enzyme activity, and muscle fibers diameters between the refeeding groups. All in vivo comparisons were performed between the groups at each experimental timepoint. The differences between timepoints were not assessed. Data were analyzed using GraphPad Prism v.6 (GraphPad Software, La Jolla, CA, USA, www.graphpad.com, accessed on 10 November 2020). The data were represented as mean ± SD. Statistical significance was considered when the *p*-value was <0.05.

## Data Availability

All the produced data is contained within this article or in Supplementary Materials.

## Supplementary Materials

The following are available online at https://www.mdpi.com/1422-0067/22/6/2995/s1.
