# Supplementary material for: Ascorbic Acid Supplementation Improves Skeletal Muscle Growth in Pacu (Piaractus mesopotamicus) Juveniles: In Vivo and In Vitro Studies
Source: Int J Mol Sci. 2021 Mar 15;22(6):2995. doi: 10.3390/ijms22062995 (PMC7998472; doi:10.3390/ijms22062995)
Supplement: Supplementary file 1 [file ijms-22-02995-s001.pdf]

International Journal of
Molecular Sciences

Supplementary Materials:

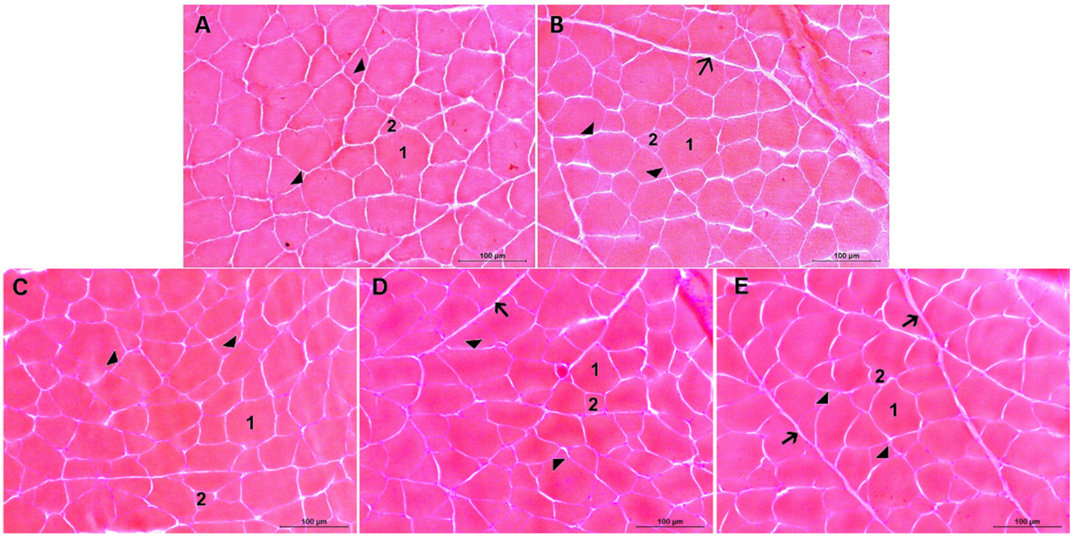

Figure S1: Representative transverse histological section from pacu skeletal muscle in (**A**) C group, continuous fed, (**B**) F group, after 15 days of fasting, and after 30 days of refeeding with AA supplementation in (**C**) H-AA (400 mg/kg of AA), (**D**) B-AA (200 mg/kg of AA), and (**E**) L-AA (100 mg/kg of AA) groups. Muscle fibers distribution in mosaic pattern with large fibers (1), interspersed with small fibers (2). Extracellular matrix forming the perimysium (arrow) and endomysium (arrowhead). Hematoxylin and Eosin staining. 20x magnification. (Bars: 100 µm). AA = ascorbic acid.

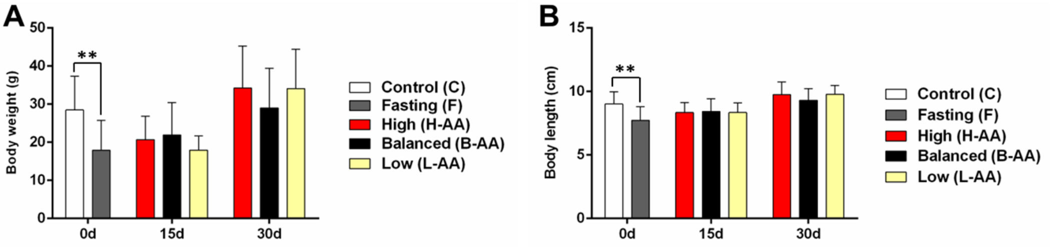

Figure S2: *In vivo* (**A**) body weight (g) and (**B**) body length (cm) of the fish continuous fed (Control, C), submitted to 15 days of fasting (F) and supplemented with 400 mg/kg (H-AA group), 200 mg/kg (B-AA group), and 100 mg/kg (L-AA group) of AA for 15 and 30 days of refeeding after 15 days of fasting. Data represented as mean ± SD. Statistical significance was determinate by Mann-Whitney between C and F groups and by one-way ANOVA followed by Tukey's posthoc test between refeeding groups (n= 6 animals/group). ** denotes $p < 0.01$. AA = ascorbic acid.

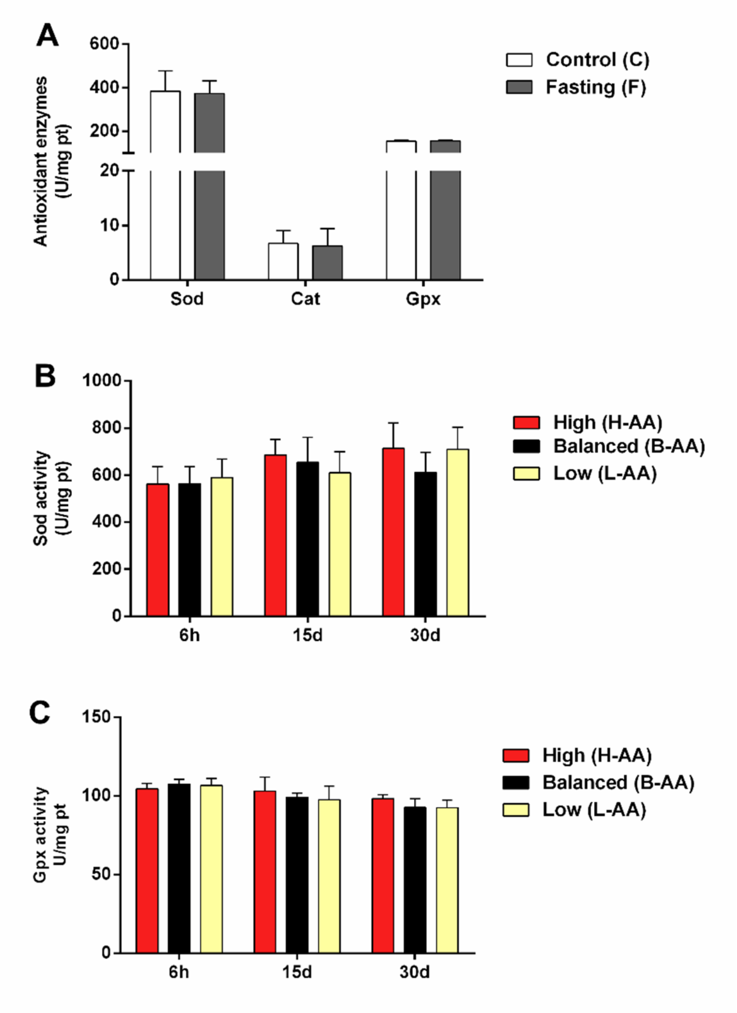

Figure S3: *In vivo* activity of Superoxide dismutase (Sod), Catalase (Cat) and Glutathione peroxidase (Gpx) in muscle tissue of fish continuous fed (C group) and submitted to 15 days of fasting (F group) and in fish supplemented with 400 mg/kg (H-AA group), 200 mg/kg (B-AA group) and 100 mg/kg (L-AA group) of AA for 6 hours, 15 and 30 days of refeeding after 15 days of fasting. Data represented as mean ± SD. No statistical difference was observed between experimental groups in each period analyzed. Statistical significance was determinate by one-way ANOVA followed by Tukey's posthoc, $p < 0.05$ ($n = 6$ animals/group). AA = ascorbic acid.

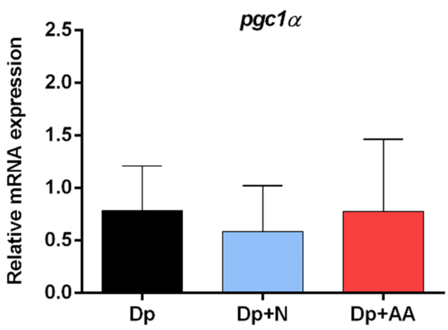

Figure S4: *In vitro* oxidative metabolism gene expression (*pgc1α*) in conditions of nutrient deprivation (Dp) for 96 hours, nutrient deprivation (72 hours) + nutrient addition (24 hours) (Dp+N) and nutrient deprivation (72 hours) + AA supplementation (24 hours) (Dp+AA). Relative mRNA expression normalized to *rpl13* expression. Fold change from the Dp group. Data represented as mean ±

SD from five independent cell isolation process. No statistical difference was observed between experimental groups. Statistical significance was determinate by one-way ANOVA followed by Tukey's posthoc test (n= 5 cell culture/group, $p < 0.05$). Dp = deprivation; N = nutrient; AA = ascorbic acid.

Table S1 – Formulation and proximate composition of the experimental diets containing low (L-AA), balanced (C/B-AA) and high (H-AA) ascorbic acid supplementation.

Ingredients (%)	Absence	H-AA	C/B-AA	L-AA
Soybean meal	43.80	43.84	43.82	43.81
Poultry by-product meal	10.00	10.00	10.00	10.00
Corn	31.50	31.35	31.42	31.46
Wheat middlings	10.00	10.00	10.00	10.00
Soybean oil	1.00	1.00	1.00	1.00
Dicalcium phosphate	2.75	2.75	2.75	2.75
BHT[1]	0.02	0.02	0.02	0.02
Premix vit/min[2]	0.63	0.63	0.63	0.63
Vitamin C[3]	-	0.1144	0.0572	0.0286
Salt	0.30	0.30	0.30	0.30
Calculated composition				
Vitamin C (mg/kg)	ns[4]	400	200	100
Digestible energy (kcal/ kg)	3060	3051	3052	3053
Digestible protein (%)	23.84	23.84	23.84	23.84
Crude protein (%)	27.10	27.10	27.10	27.10
Crude fiber (%)	4.20	4.20	4.20	4.20
Ether extract (%)	4.82	4.82	4.82	4.82

[1]Butyl-Hydroxy-toluene.
[2]Vitamin and mineral premix (kg of product): Vit. A = 1.200.000 UI; vit. D3 = 200.000 UI; vit. E = 12.000 mg; vit. K3 = 2.400 mg; vit. B1 = 4.800 mg; vit. B2 = 4.800 mg; vit. B6 = 4.000 mg; vit. B12 = 4.800 mg; folic acid = 1.200 mg; calcium pantothenate = 12.000 mg; biotine = 48 mg; choline =65.000 mg; nicotinic acid = 24.000 mg; Fe = 10.000 mg; Cu = 600 mg; Mn = 4.000 mg; Zn = 6.000 mg; I = 20 mg; Co = 2 mg e Se = 20 mg.
[3]Vitamin C Rovimix Stay-C® 35. DSM Nutritional Products. Switzerland. Values calculated considering 35% of the AA activity.
[4]ns: not supplemented.

Table 2. – Primers used in the RT-qPCR analyses.

Gene symbol	Gene name	Primer sequence
igf1	*insulin-like growth factor 1*	L: ATTTCAGCAAGCCAACAGGT<br>R: CGCACAATACATCTCAAGTCG
mtor	*serine/threonine-protein kinase TOR*	L: TTGGGAGAGACGTACTGC<br>R: CACAGGACTGGTGTAGGAA
rragc	*ras-related GTP-binding*	L: GCAACTGAGGGATGAGCTTC<br>R: CACCAGCGAGCTAATGATGA
rps6kb1a	*ribosomal protein S6 kinase 1a*	L: ATGCTCTCTGTGCCTTGGT<br>R: CCGACGAAGAACTTGAGGAG
mafbx	*muscle atrophy F-box protein/ F-box protein 32*	L:TCTTTGGTGCTCCCCTTGTG<br>R: TAAAACCGAGGACGGCTGG
murf1a	*muscle-specific ring finger protein 1/*	L: ATGTTGCTGTTGTCCATACTCTG<br>R: ATCACATCACCCAGGAGCA

	tripartite motif-containing 63a	
fbxo25	*f-box protein 25*	L: CGTAGGTGAAGTAGCCGCCA<br>R: GGAGAAGTCCAGCCTGTTGA
pcna	*proliferating cell nuclear antigen*	F: TGTCCCAGACCAGCAATGTG<br>R: GCTGAGCGTGACTGTTTTGG
myod	*myoblast determination protein 1 homolog*	L: GGGTCGTCGTAGAAGTCGTC<br>R: TTTTTGGTCGGAAGAGATGG
myog	*myogenin*	L: CAGACCAGAGGTTTTATGAA<br>R: TAGATGTTGGGGATGGCTTG
pgc1-α	*peroxisome proliferator-activated receptor gamma coactivator 1-alpha*	L: GAGGGTGAGCGTTCAAAGAG<br>R: ATGAGGCTGAGCAGAGAGGA
sdha	*succinate dehydrogenase [ubiquinone] flavoprotein subunit a*	L: ACCTGATGCTGAATGCTGTG<br>R: AGTGTGCTTCCTCCAGTGC
rpl13	*60S ribosomal protein L13*	L: ATCAACAGGAAAGTAGCCC<br>R: AGGATGAGTTTGGAGCGGTA

L: left strand; R: right strand.
